# Dual regulation by microRNA-200b-3p and microRNA-200b-5p in the inhibition of epithelial-to-mesenchymal transition in triple-negative breast cancer

**DOI:** 10.18632/oncotarget.3184

**Published:** 2015-03-21

**Authors:** Lyndsay V. Rhodes, Elizabeth C. Martin, H. Chris Segar, David F. B. Miller, Aaron Buechlein, Douglas B. Rusch, Kenneth P. Nephew, Matthew E. Burow, Bridgette M. Collins-Burow

**Affiliations:** ^1^ Department of Medicine-Section of Hematology and Medical Oncology, Tulane University, New Orleans, LA, USA; ^2^ Department of Pharmacology, Tulane University, New Orleans, LA, USA; ^3^ Medical Sciences and Department of Cellular and Integrative Physiology, Indiana University School of Medicine, Bloomington, IN, USA; ^4^ Department of Biological Sciences, Florida Gulf Coast University, Fort Myers, FL, USA; ^5^ Indiana University Center for Genomics and Bioinformatics, Bloomington, IN, USA

**Keywords:** triple negative breast cancer, RHOGDI, miRNA biogenesis, star strand, isomiRs

## Abstract

Epithelial to mesenchymal transition (EMT) involves loss of an epithelial phenotype and activation of a mesenchymal one. Enhanced expression of genes associated with a mesenchymal transition includes ZEB1/2, TWIST, and FOXC1. miRNAs are known regulators of gene expression and altered miRNA expression is known to enhance EMT in breast cancer. Here we demonstrate that the tumor suppressive miRNA family, miR-200, is not expressed in triple negative breast cancer (TNBC) cell lines and that miR-200b-3p over-expression represses EMT, which is evident through decreased migration and increased CDH1 expression. Despite the loss of migratory capacity following re-expression of miR-200b-3p, no subsequent loss of the conventional miR-200 family targets and EMT markers ZEB1/2 was observed. Next generation RNA-sequencing analysis showed that enhanced expression of pri-miR-200b lead to ectopic expression of both miR-200b-3p and miR-200b-5p with multiple isomiRs expressed for each of these miRNAs. Furthermore, miR-200b-5p was expressed in the receptor positive, epithelial breast cancer cell lines but not in the TNBC (mesenchymal) cell lines. In addition, a compensatory mechanism for miR-200b-3p/200b-5p targeting, where both miRNAs target the RHOGDI pathway leading to non-canonical repression of EMT, was demonstrated. Collectively, these data are the first to demonstrate dual targeting by miR-200b-3p and miR-200b-5p and a previously undescribed role for microRNA processing and strand expression in EMT and TNBC, the most aggressive breast cancer subtype.

## INTRODUCTION

Breast cancer can be classified into distinct sub-types, including luminal and basal subtypes, based on molecular markers that define their phenotype and predict therapeutic response [1]. The triple-negative sub-type is characterized by loss of estrogen receptor, progesterone receptor, and Her2/Neu, and therefore is not responsive to current targeted therapeutics. Triple-negative breast cancer (TNBC) is also one of the most aggressive subtypes of breast cancer, with a clinically observed higher rate of distant metastasis and poor overall survival [1, 2]. TNBC can be further subdivided into 6 subtypes based upon gene expression profiles, and include basal-like (1 and 2), immunomodulatory, mesenchymal, mesenchymal stem-like, and luminal androgen receptor [3], illustrating the heterogeneity of this subtype. Although molecular subtyping has begun to elucidate the signaling events regulating this aggressive form of breast cancer, much remains to be investigated. RNA based next generation deep sequencing for both large and small RNAs allows for great depth in investigating differences in gene expression profiles for breast cancer subtypes.

miRNA mediated regulation of breast cancer has received considerable attention due to evidence of miRNA regulation of drug resistance, metastasis, receptor status, and the epithelial-to-mesenchymal transition (EMT) [4–6]. The expression of the miR-200 family cohort of miRNAs is lost in mesenchymal breast cancer subtypes compared to epithelial subtypes [7]. This miRNA family exists in two clusters: one on chromosome 1 (miR-200b-3p, miR-200b-5p, miR-200a-3p, miR-200a-5p, and miR-429) and the other on chromosome 12 (miR-200c-3p, miR-200c-5p, miR-141-3p, and miR-141-5p) [8, 9]. Conventionally the miR-200 family is a regulator of epithelial–cadherin (CDH1) expression through the direct targeting of zinc finger E-box-binding homeobox 1 (ZEB1) and 2 (ZEB2) [10]. However, canonical signaling of miR-200 family and ZEB1/2 demonstrates an inhibitory mechanism where ZEB1 and ZEB2 can repress miR-200 family expression [11]. Collectively a negative feedback loop has been established for the miR-200 family and ZEB1/2 which ultimately leads to the regulation of CDH1 and EMT/MET. The increased expression of ZEB1/2 leads to the loss of miR-200 and CDH1, while the increased expression of miR-200 leads to loss of ZEB1/2 and increased CDH1 [11]. Although this has been the dogma for miR-200 regulation of EMT, recent studies suggest non-canonical mechanisms for miR-200 family regulation of EMT independent of ZEB1 and ZEB2, as well as miR-200 family as regulators of EMT through both canonical and non-canonical ZEB/miR-200/CDH1 mechanisms and through interactions with TGF-β, hedgehog, and RHO signaling pathways [12–14].

Here we demonstrate through next generation sequencing analysis miR-200b-3p regulation of EMT in the TNBC subtype through inhibition of the RHO signaling cascade. Additionally, through the enforced expression of pri-miR-200b we demonstrate altered processing and enhanced expression of miR-200b-5p. Furthermore we demonstrate a compensatory mechanism where both miR-200b and miR-200b-5p target the RHO signaling cascade. These results demonstrate a novel mechanism for miR-200 family in the regulation of EMT, highlighting the importance of miRNA processing and miRNA strand expression in breast cancer.

## RESULTS

### MiR-200 family is differentially expressed across breast cancer cell lines

**Figure 1:**
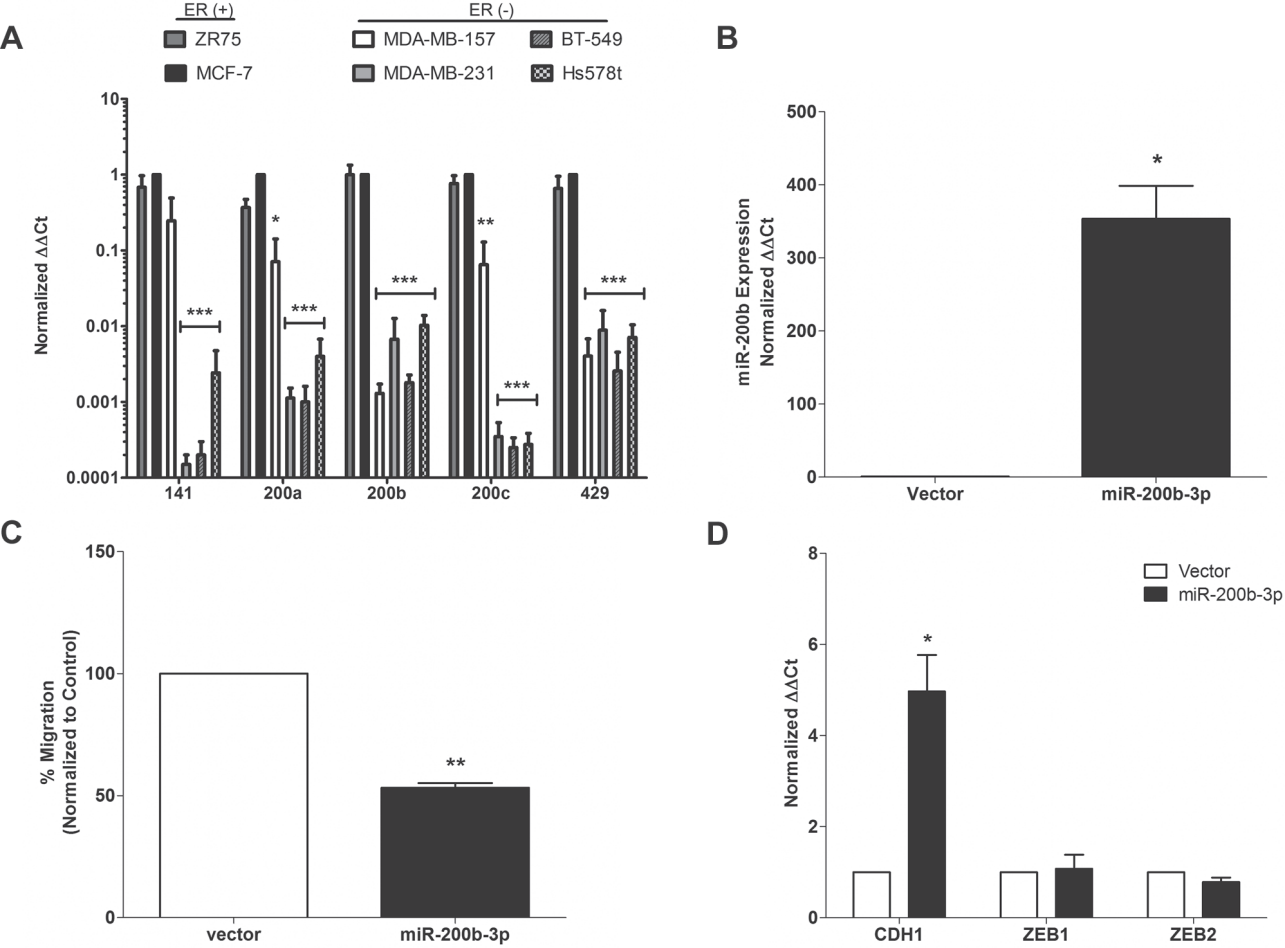
miR-200b-3p regulates epithelial to mesenchymal transition in triple negative breast cancer **(A)** A cohort of ER^+^ (ZR75, MCF-7) and ER^−^ (MDA-MB-157, MDA-MB-231, BT-549, Hs578t) breast cancer cell lines were tested by qPCR to determine basal miR-200b-3p expression. U6 was used for internal normalization and compared to the MCF-7 breast cancer cell line (designated as 1). **(B)** qPCR for stable expression of miR-200b-3p in the MDA-MB-231 cell line following transfection with the pLEmiR-tRFP-miR-vector or –pri-miR-200b expression plasmids. **(C)** Transwell migration assay of MDA-MB-231-vector and –miR-200b cell lines following 24 hours. Fixed and stained membranes were visualized by microscopy for cell migration and graphs represent mean number of migrated cells per field of view. **(D)** qPCR for basal CDH1, ZEB1, and ZEB2 expression in the MDA-MB-231-vector cell line versus –miR-200b. Beta-actin was used as loading control and compared to MDA-MB-231-vector cell line (designated as 1). For all experiments error bars represent SEM for *n* = 3, and significance denoted by *p < 0.05, **p < 0.01, ***p < 0.001.

qPCR analysis of a panel of breast cancer cell lines revealed suppressed expression of all miR-200 family members in cell line models of the TNBC subtype (Figure 1A). However, only repression (~100-fold) of miR-200b-3p was consistently observed in all TNBC cell lines tested. While there has been a clear association of miR-200 members in regulation of EMT, cell motility and metastasis, the role of miR-200b-3p in this process has not been fully established [15, 16]. As a recent report using computational modeling of miRNA and mRNA expression data from patient samples demonstrated a loss of miR-200b-3p expression and activity that specifically correlated to the basal breast cancer sub-type [17], we chose to further examine the specific effects of miR-200b-3p on the TNBC sub-type.

### miR-200b-3p expression suppresses TNBC cell migration and EMT-gene expression

To directly assess the sub-type specific effects of miR-200b-3p, the TNBC cell line, MDA-MB-231, was transfected with pLEmiR-tRFP-miR-vector or –pri-miR-200b expression plasmids. Following stable selection, expression of mature processed miR-200b-3p was confirmed by qPCR. Expression of mature miR-200b-3p increased 353.5-fold in MDA-MB-231 cells (Figure 1B), compared to corresponding vector control cell lines.

As previous studies have shown that miR-200 family regulates the metastatic potential of cancer cells [12], we examined the effect of miR-200b expression on TNBC cell migration using *in vitro* transwell migration assays, which have been correlated with metastatic potential [18]. MDA-MB-231-miR-200b cells showed a 50% reduction in migration capacity than their vector counterparts (Figure 1C), indicating that miR-200b-3p plays a role in migration suppression and in agreement with a previous study [19].

The EMT has been recognized as a major player in cancer cell migration, invasion, and metastasis [20]. EMT is characterized by the loss of epithelial cell markers, namely CDH1, enhanced expression of mesenchymal cell markers, including neuronal–cadherin (CDH2) and vimentin (VIM), and increased expression of CDH1 transcriptional repressors such as ZEB1 and ZEB2 [21–24]. The expression of EMT markers, including CDH1, CDH2, VIM, ZEB1 and ZEB2, is associated with poor prognosis of breast cancer patients [25, 26]. As such, regulators of EMT have become attractive targets for the development of anti-metastasis therapies. The canonical suppression of EMT by miR-200 family members occurs through targeting of ZEB1 and 2, direct repressors of the epithelial marker, CDH1 [10, 27, 28]. To better assess the effects of miR-200b-3p overexpression in the MDA-MB-231-miR-200b cell line qPCR for CDH1, ZEB1, and ZEB2 was performed. Results demonstrated a significant increase in CDH1 expression in the MDA-MB-231-miR-200b cells compared to vector control but expression of ZEB1 and ZEB2 remained essentially unchanged (Figure 1D).

### miR-200b expression induces global gene expression changes

**Figure 2:**
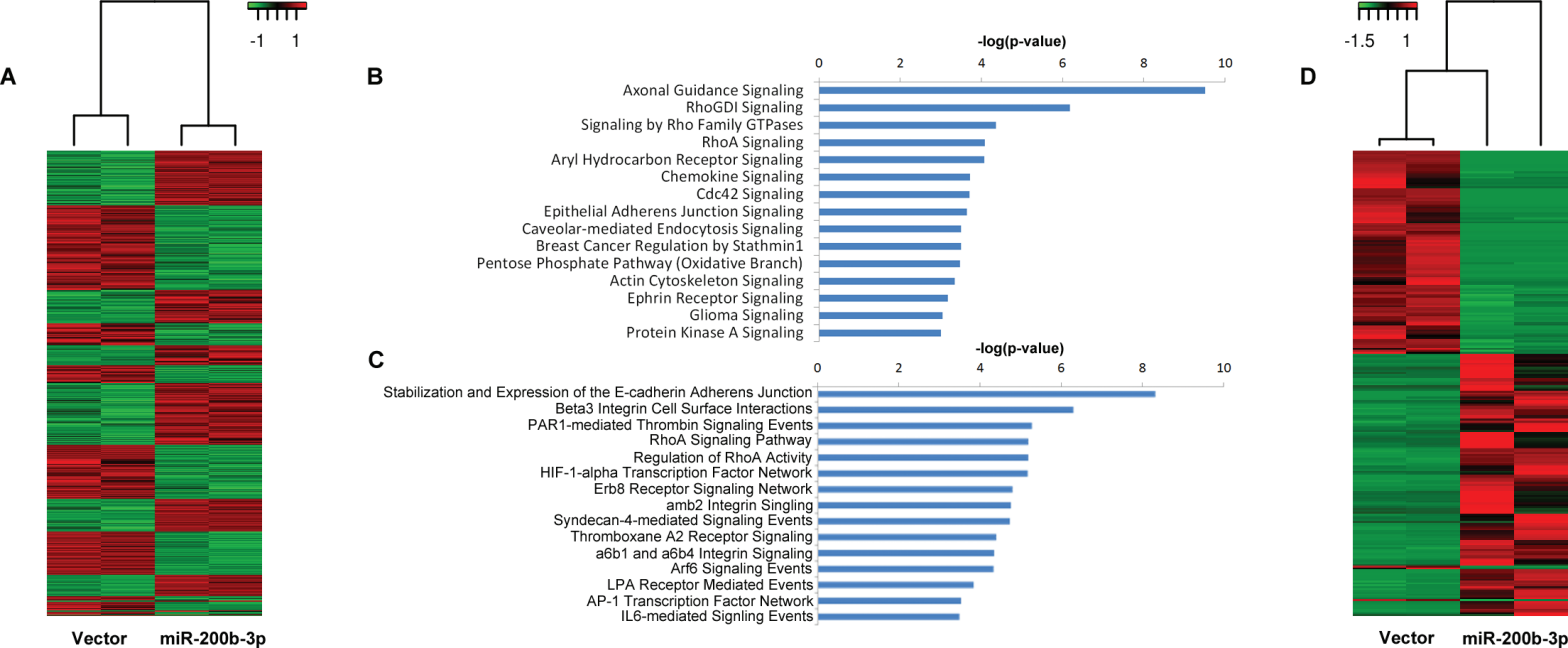
Overexpression of miR-200b-3p Induces global gene expression changes in the MDA-MB-231 cell line **(A)** Heat map representation of global RNA expression changes in the MDA-MB-231-miR-200b cell line versus –vector. **(B)** and **(C)** represent pathways associated with genes that are significantly downregulated (B) or upregulated (C) in the MDA-MB-231-miR-200b cell line compared to –vector control. Pathway analysis was performed using QIAGEN's Ingenuity^®^ Pathway Analysis (IPA). **(D)** Heat map representation of global miRNA expression changes in the MDA-MB-23-miR-200b cell line versus –vector.

Due to the lack of ZEB1 and ZEB2 repression in MDA-MB-231-miR-200b cell line, we evaluated global transcriptome changes invoked through pri-miR-200b overexpression using next generation RNA-sequencing of the MDA-MB-231-miR-200b versus vector control cells. Prior to sequencing, RNA libraries were fractioned by size to generate full length and small RNA libraries, which were analyzed independently. Gene expression analysis of the full length data set revealed expression levels of 1831 genes that were significantly (* p* < 0.05) altered following pri-miR-200b overexpression, with 824 genes upregulated and 1007 genes downregulated (Figure 2A). Some genes associated with EMT were demonstrated to be both significantly enhanced and repressed (Supplemental Table 1). However, these genes showed only minimal changes in expression level. Pathways significantly downregulated in the MDA-MB-231-miR-200b cells included many pathways associated with cell motility and cancer metastasis, such as axonal guidance, chemokine, epithelial adherens junction, and actin cytoskeleton signaling (Figure 2B). Of particular interest was the down-regulation of RHO signaling, given the widely known role of RHO dysregulation in cancer [29]. On the other hand, the most upregulated pathway in the miR-200b stable cells was the stabilization and expansion of E-cadherin adherens junction (Figure 2C), a critical pathway known to be deregulated in invasive cancer [30, 31] and a key player in EMT [23]. Interestingly, classical EMT signaling events were not predicted to be regulated in our 200b cells by pathway analysis. Overall, pathway analysis of these up and down regulated genes indicates inhibition of the invasive phenotype of TNBC by miR-200b expression, through non-canonical signaling.

Analysis of the small RNA fraction revealed significant alteration in expression of 1363 transcripts, of which 503 were upregulated and 859 were downregulated (Figure 2D). Interestingly, only 15 miRNA were included in these significantly altered transcripts, including miR-200b-3p (Table 1).

**Table 1:** microRNA with significantly altered expression in MDA-MB-231-miR-200b cells compared to vector control

miRNA	Fold change	Role in cancer	Refs
103a-2	0.31	Not known	
181b1	0.02	oncomiR	[65]
1915	0.00005	Tumor suppressor, Targets Bcl-2	[66]
20a	38.49	oncomIR. miR-17-92 family	[67]
200b	5750.37	Anti-migratory, anti-EMT	[19, 50]
21	0.36	oncomiR	[68]
221	0.15	oncomiR, enhanced invasion	[69]
3074	0.23	Not known	
33B	0.06	Regulation of metabolism and cell cycle	[70, 71]
3618	3.00	Not known	
3648	0.35	Not known	
574	0.05	Not known	
598	0.12	Associated with esophageal cancer	[72]
663a	0.26	Not known	
let7f1	4.30	Anti-invasive, anti-metastatic	[73]

### RNA-sequencing indicates differential regulation of gene expression by miR-200b-3p and miR-200-5p

**Figure 3:**
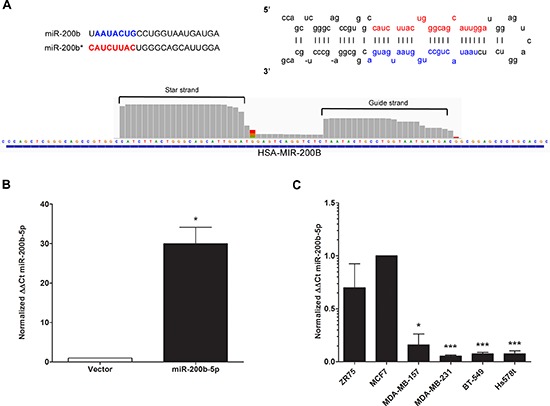
miR-200b-5p is differentially regulated between ER+ and triple negative breast cancer subtypes **(A)** Representative graphical analysis of reads for miR-200b-3p and miR-200b-5p expression in the MDA-MB-231-miR-200b cell line as viewed in Integrative Genomics Viewer (IGV). **(B)** qPCR for stable expression of miR-200b-3p in the MDA-MB-231 cell line following transfection with the pLEmiR-tRFP-miR-vector or –pri-miR-200b expression plasmids. U6 internal normalization and comparison to the MDA-MB-vector (designated as 1). **(C)** A cohort of ER^+^ (ZR75, MCF-7) and ER- (MDA-MB-157, MDA-MB-231, BT-549, Hs578t) breast cancer cell lines were extracted for qPCR to determine basal miR-200b-5p expression. Normalization was to U6 and the MCF-7 breast cancer cell line designated as 1 for comparison of relative expression. For all experiments error bars represent SEM for *n* = 3, and significance expressed as *p < 0.05, ***p < 0.001.

Since there was no observed significant change in ZEB1 or ZEB2 in our MDA-MB-231-miR-200b cell line, we next sought to determine if any alterations were made to the miR-200b-3p transcript. As miR-200b-3p was induced as a precursor miRNA, alterations to the 5′ end of the miRNA may have been induced during maturation. This would then account for the loss of ZEB targeting. Surprisingly, upon validation of miR-200b expression in our whole transcriptome analysis as observed in the Integrative Genomics Viewer (IGV), expression of both the mature miR-200b-3p (guide strand) and miR-200b-5p (the star strand/passenger) was enhanced in MDA-MB-231-miR-200b (Figure 3A). Additionally there was no observed loss of 5′ heterogeneity in either miRNA strand (Figure 3A). Recent reports have shown that miRNA processing can favor strand selection, with the star strand or passenger strand being expressed at equal or higher levels than the conventional guide strand [32, 33]. Additionally, isomiRs, a miRNA transcript that differs from the primary transcript by one or two nucleotides, exist and demonstrate altered expression between cellular systems [34–36]. Since our miR-200b construct was expressed as pri-miR-200b, we evaluated previously published deep sequencing data to determine if miR-200b-5p can exist with physiologically relevant expression levels or if our enhanced miR-200b-5p expression was merely an artifact of enforced expression. As seen in miRBase, miR-200b-5p isomiRs are documented and can occur at physiologically relevant levels [37–40] and can be expressed at detectable levels (data not shown) [41–45]. qPCR validation for miR-200b-5p in our MDA-MB-231-miR-200b over expressing cell line revealed a significant increase in miR-200b-5p expression levels (Figure 3B). To examine if miR-200b-5p has a significant role in TNBC, we then examined miR-200b-5p expression across a panel of breast cancer cell lines. Similar to the pattern observed in miR-200b-3p expression (Figure 1A), miR-200b-5p demonstrated substantially reduced expression in TNBC versus ER^+^/luminal cell lines (Figure 3C). Although the -5p strand by convention represents the previously designated star strand is generally thought to have low to no basal expression, the relative expression of miR-200b-5p in the ER^+^ cell lines tested was at physiologically robust levels (similar to that of miR-200b-3p; Figure 1A).

Since miR-200b-3p and miR-200b-5p were both expressed in our MDA-MB-231-miR-200b cell line and expression of both -3p and -5p were observed at physiologically relevant and similar endogenous levels in ER^+^ breast cancer cell lines, we next sought to evaluate the preference for isomiR expression for both of these miRNAs in our deep sequencing analysis. Relative counts of expressed isomiRs for both miRNAs are shown in Table 2. Notably, miR-200b-3p demonstrates greater variance on the 3′ end than miR-200b-5p, and as stated above, there were little alterations in the 5′ end of either miRNA.

**Table 2:** Prevalence of miR-200b-3p and miR-200b-5p IsomiRs in MDA-MB-231-miR-200b Cell Line

hsa-miR-200b-5p	hsa-miR-200b-3p	Count
**CAUCUUACUGGGCAGCAUUGGA**		64
CAUCUU**C**CUGGGCAGCAUUGGA		1
CA**C**CUUACUGGGCAGCAUUGGA		1
CAUCUUACUGGGCAGCAUUG		3
CAUCUUACUGGGCAGCAUU**U**		1
CAUCUUACUGGGCAGCAUUGG		19
CAUCUUACUGGGCAGCAUUG**U**		2
CA**C**CUUACUGGGCAGCAUUGG		1
CAUCUUACUGGGCAGCAUU		8
CAUCUUACUGGGCAGCAU		1
CAUCUUACUGGGCAGCAUUGGAU		11
CAUCUUACUGGGCAGCAUUGGA**G**		1
CAUCUUACUGGGCAGCAUUGGA**A**		1
CAUCUUACUGGGCAGCAUUGGAU**U**		17
CAUCUUACUGGGCAGCAUUGGAU**A**		4
CAUCUUACUGGGCAGCAUUGG**G**U**A**		1
AUCUUACUGGGCAGCAUUGGA		1
AUCUUACUGGGCAGCAUUG		1
AUCUUACUGGGCAGCAUUGGAU		6
	**UAAUACUGCCUGGUAAUGAUGA**	3
	UAAUAC**G**GCCUGGUAAUGAUGA	1
	UAAUACUGCCUGGUAAUGA**C**GA	1
	UAAUACUGCCUGGUAA**A**GAUGA	1
	UAAUACUGCCUGGUA**C**UGAUGA	1
	UAAUACUGCCUGGU**G**CUGAUGA	1
	U**G**AUACUGCCUGGUAAUGAUGAC	4
	UAAUACUGCCUGGU**G**AUGAUGAC	5
	UAAUACUGCCUGGUAAUGA**G**GAC	2
	UAAUACUGCCUGGUAA**A**GAUGAC	4
	UAAUACUGCCUGGUAA**G**GAUGAC	8
	UAAUACUGCCUGGUAA**C**GAUGAC	2
	UAAUACUGCCUGGUA**G**UGAUGAC	2
	UAAUACUGCCUGGUAAU**A**AUGAC	1
	UAAUACUGCCUGGUA**C**UGAUGAC	1
	UAAUACUGCCUGGUAA**G**GAUGAC**A**	1
	UAAUACUGCCUGGUAA**G**GAUGAC**C**	1
	UAAUACUGCCUGGUA**G**UGAUGAC**U**	1
	UAAUAC**G**GCCUGGUAAUGAUGAC**U**	1
	AAUACUGCCUGGUAUU**C**AUGAC**U**	1
	**A**AUACUGCCUGGUAUU**C**AUGAC**U**	1
	UAAUACUGCCUGGUAAUGA**AT**	6
	UAAUACUGCCUGGUAAUG	11
	UAAUACUGCCUGGUAAU	17

Colored text indicates primary sequence for miR-200b-5p (red) and miR-200b-3p guide (blue). Black text represents isomiR sequences with altered nucleotides indicated in bold/underlined print.

**Figure 4:**
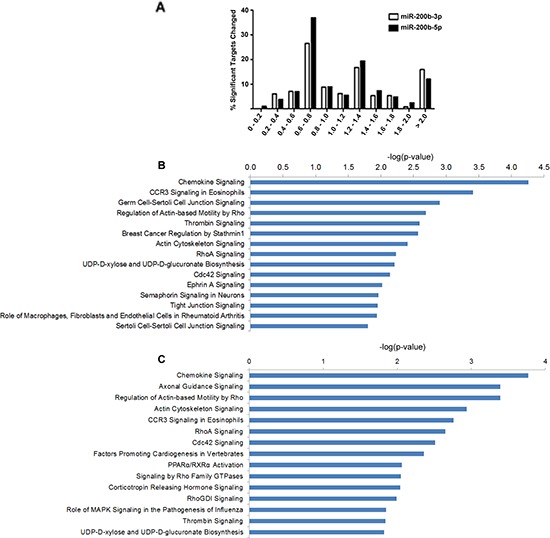
miR-200b-3p and miR-200b-5p display synonymous regulation patterns of target pathways Deep sequencing analysis for gene fold change and altered pathways in the MDA-MB-231-miR-200b cell line. **(A)** Graphical representation of observed fold change of genes which contain either a miR-200b-3p or miR-200b-5p seed site with significantly altered expression by deep sequencing. **(B) and (C)** represent top pathways predicted to be affected by genes which contain either a miR-200b-3p (B) or miR-200b-5p (C) seed site and were observed to be significantly down regulated. Pathways were determined through the use of IPA (Ingenuity^®^ Systems, www.ingenuity.com).

To evaluate the effects of miR-200b-3p and miR-200b-5p on gene expression we analyzed expression changes in genes predicted to have a seed site to either miR-200b-3p or miR-200b-5p. Seed sites for miR-200b-3p and miR-200b-5p were generated using an in house algorithm, Seedfinder, to identify all 7-mer and 8-mer seeds throughout the human genome; from this data seed sites found in the 3ʹUTR of transcripts were analyzed. As seen in Figure 4A, miR-200b-3p and miR-200b-5p significantly repressed predicted target genes similarly, with only a slight increase in the repression of miR-200b-5p targets. Unexpectedly, our deep sequencing analysis revealed that some miRNA targets were not repressed. Furthermore some predicted miR-200-5p and miR-200b-3p targets demonstrated significantly enhanced expression. Due to this, we next chose to evaluate alterations in the MDA-MB-231-miR-200b transcriptome to better determine a mechanism for loss of targeting by miR-200b-3p/200b-5p in the MDA-MB-231 cell line. Predicted targets were evaluated for alterations in mRNA 3ʹUTR architecture and mRNA isoform variance. Due to the large number of predicted targets that were not changed or increased in our MDA-MB-231-miR-200b cell line, genes demonstrating low basal levels of expression or those that did not contain an 8-mer miR-200b-3p/200b-5p seed site were not evaluated. Table 3 demonstrates the mRNAs that were predicted to be targeted by miR-200b-3p or miR-200b-5p with increased expression levels and describes if alterations in mRNA 3ʹUTR or isoform expression was observed. Interestingly, many of the genes predicted to be targeted by miR-200b-3p or miR-200b-5p demonstrated loss of 3ʹUTR. Some EMT associated genes demonstrating loss of 3ʹUTR include DUSP1, ROCK2, DLC1, FZD3, AHR, and ELK4.

**Table 3:** Loss of 3ʹUTR in genes with miR-200b-3p and miR-200b-5p seed sites

	miR-200b-3p				miR-200b-5p		
Target gene	Expression	3′UTR Loss	Fold Change	Target gene	Expression	3′UTR Loss	Fold Change
ABI2	Unchanged	No		AHR	Unchanged	Yes	
BAP1	Unchanged	No		ALG2	Unchanged	Yes	
CAST	Unchanged	Yes		AQR	Unchanged	Yes	
COX7C	Increased	Yes	1.33 (*p* < 0.01)	BTBD1	Increased	Yes	1.37 (*p* < 0.05)
CYTH3	Unchanged	No		CHSY1	Unchanged	Yes	
DLC1	Increased	Yes	1.97 (*p* < 0.001)	CPNE3	Increased	Yes	1.27 (*p* < 0.05)
DOCK4	Unchanged	Yes		EPT1	Unchanged	No	
DUSP1	Increased	Yes	3.89 (*p* < 0.001)	FZD3	Increased	Yes	1.94 (*p* < 0.001)
ELK4	Unchanged	Yes		HNRNPU	Unchanged	Yes	
ELL2	Unchanged	Yes		KCTD12	Unchanged	Yes	
HS3ST1	Increased	Yes	2.06 (*p* < 0.01)	LAP3	Unchanged	Yes	
JUN	Unchanged	No		NAMPT	Unchanged	Yes	
KIF1B	Unchanged	Yes		ORC2	Unchanged	Yes	
RNGTT	Unchanged	Yes		PCMTD2	Unchanged	Yes	
ROCK2	Unchanged	Yes		PPRC1	Increased	Yes	1.52 (*p* < 0.001)
RRP15	Unchanged	Yes		RAP2A	Unchanged	No	
TRIM33	Unchanged	Yes		SDPR	Unchanged	Yes	
UBE2B	Unchanged	Yes		UBE3A	Unchanged	No	
USP46	Unchanged	Yes		UHMK1	Unchanged	No	
WDR91	Unchanged	No		UHRF1BP1	Unchanged	Yes	
WIPF1	Unchanged	Yes		USP22	Unchanged	Yes	
ZMAT3	Increased	Yes	1.44 (*p* < 0.05)	WDFY3	Unchanged	Yes	

### miR-200b-3p and miR-200b-5p synergize to target non-canonical EMT pathway

To gain greater insight into miR-200b induced alterations in gene expression, all predicted miR-200b-3p and miR-200b-5p targets which were significantly down regulated in our deep sequencing analysis were analyzed through the use of IPA (Ingenuity^®^ Systems, www.ingenuity.com). Top down regulated pathways for miR-200b-3p (Figure 4B) and miR-200b-5p (Figure 4C) included many overlapping pathways reported in our original pathway analysis of all significantly down regulated genes (Figure 2B). Interestingly, EMT signaling events were not predicted by pathway analysis of significantly down regulated predicted targets. Overall, given our biological data demonstrating an increase of the MET regulating gene, CDH1, and a loss of cellular migration *in vitro*, along with pathway analysis of transcriptome changes suggest that miR-200b-3p/200b-5p act to inhibit the invasive phenotype of TNBC through non-canonical signaling. Of interest was the repeated evidence of inhibition of the RHO signaling pathway evident by pathway analysis of down regulated target genes for both miR-200b-3p and miR-200b-5p.

**Figure 5:**
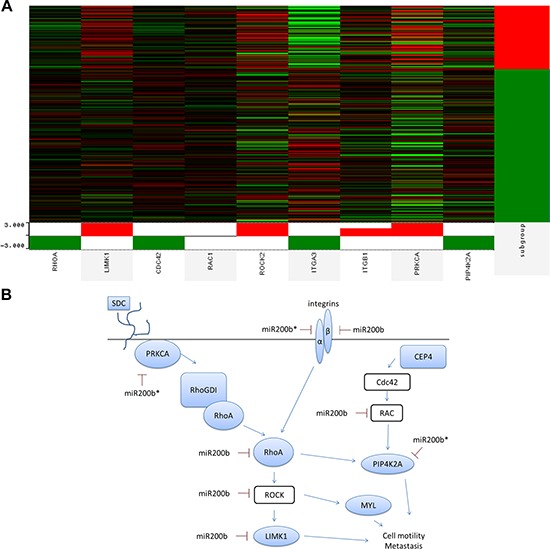
miR-200b-3p and miR-200b-5p synergize to target non-canonical EMT pathway in triple negative breast cancer tumors **(A)** Deep sequencing data of human breast cancer tumors was obtained from TCGA data portal and was analyzed for genes associated with the RHOGDI pathway (RHOA, LIMK1, CDC42, RAC1, ROCK2, ITGA2, ITGA1, PRKCA, PIP4K2A) with respect to the basal subtype. Heat map depicts high expression (red, +1) and low expression (green, −1). Platform analyzed was TCGA breast invasive carcinoma gene expression Illumina HiSeq, *n* = 1032. **(B)** Depiction of RHOGD1 pathway and co-repression of predicted miR-200b-3p and miR-200b-5p target genes.

To determine relevance of miR-200b-3p/200b-5p targeting of the RHOGDI signaling pathway in the TNCB phenotype, we performed analysis of previously published patient tumor data freely available through The Cancer Genome Atlas (TCGA) in collaboration with the UCSC Cancer Browser [46–49]. The genes RHOGDI associated genes RHOA, LIMK1, CDC42, RAC1, ROCK2, ITGA2, ITGA1, PRKCA, PIP4K2A were analyzed. These genes were chosen based on predicted targets of miR-200b-3p/200b-5p which demonstrated significantly repressed expression in our deep sequencing data or based on relevance to RHOGDI pathway. Of interest PRKCA demonstrated significantly higher levels of expression in basal like tumors and is a miR-200b-5p predicted target (Figure 5A). To better evaluate the differences between PRKCA and the triple negative phenotype, qPCR was performed for PRKCA across ER^+^ and triple negative breast cancer cell lines. PRKCA demonstrated no significant change in expression levels between the two cohorts (Supplemental Figure 1). The relevance of interactions between PRKCA, miR-200b, and EMT has been recently evaluated and there is an emerging importance of this pathway in relation to EMT in TNBC [19, 50]. Importantly, we demonstrate that key components of the RHOGDI pathway demonstrate loss of 3ʹUTR (DLC1, ROCK2) in the MDA-MB-231 cell line. This may emerge as an important factor for consideration when developing methods for intervention.

## DISCUSSION

microRNAs are key regulators of breast cancer progression where alterations in their expression can give rise to EMT and metastasis. While conventional studies on miRNAs demonstrate alterations in miRNA expression correlating to an enhanced breast cancer phenotype, the utilization of deep sequencing adds greater depth to changes in miRNA expression. Recent evidence suggests that the miRNA is more complex than previously thought. A single miRNA transcript has the potential to give rise to multiple isomiRs which have differences in expression in both the guide and star strand. Here we demonstrate that the ectopic expression of the pri-miR-200b gives rise to both miR-200b-3p and miR-200b-5p in addition to altered isomiRs for each miRNA, where both miR-200b-3p and miR-200b-5p demonstrated 3ʹheterogenity. In fact, the more prominent transcript of miR-200b-3p expressed in our MDA-MB-231 cell line is truncated at the 3′ end. The effects of miRNA 3ʹheterogenity on biological targeting and miRNA expression remain to be determined and warrant further investigation. Interestingly, while miR-200b-3p is expressed in isomiRs of variable length, there is greater consistency in the expression of the less-conventional miR-200b-5p isomiR. Mechanisms for this may be due to differences in the proteins associated with miRNA biogenesis, such as the argonautes. There are four argounatue (AGO) proteins in humans AGO1–4 and evaluation of alterations in AGO1–4 gene expression across breast cancer cell lines and tumor samples has shown that AGO2 is increased in ER^−^ samples versus ER^+^ while there is no change in AGO1, AGO3, or AGO4 [51]. Additionally the AGO proteins select for miRNA isomiRs and this selection is based on changes in both 5′ and 3′ heterogeneity [52]. Evaluation of changes in AGO expression and the implications on interactions with tumor suppressive miRNAs such as the miR-200 family may give greater insight into differences in miRNA expression changes in TNBC. There were few alteration to the 5ʹends of the miR-200b-3p and miR-200b-5p isomiRs. As the 5ʹend of the miRNA dictates the possible mRNA targets, this is important as it suggests that conventional mRNA targets of miR-200b-3p and miR-200b-5p should be repressed. Despite intact seed sequence in our miRNAs, few predicted targets of both miR-200b-3p and miR-200b-5p are repressed in the MDA-MB-231-200b cell line. Further analysis of predicted targets of miR-200b-3p and miR-200b-5p demonstrated a loss of targeting due to shortening of 3ʹUTR in the target mRNA. Actively proliferating cells and cancers demonstrate loss of 3ʹUTR through increased alternative polyadenylation (APA) [53, 54]. Shortening of the 3ʹUTR will result in loss of gene targeting by miRNAs as there are fewer seed sites present in the 3ʹUTR. Pri-miR-200b transfection into the mesenchymal MDA-MB-231 cell line resulted in loss of repression of many miR-200b-3p and miR-200b-5p targets. This evaluation draws to light considerations that must be made when evaluating treatments for breast cancer. New predictions for therapies suggest the modulation of miRNAs and demonstrate increased miRNA expression following drug treatment [55]. However our study suggests that the mRNA transcriptome must also be evaluated for miRNA modulation to be effective. Loss of 3ʹUTR in TNBC may dampen the effect of miRNA intervention due to loss of available seed-sites. In conjunction with previous studies on the miR-200 family, we demonstrate loss of miR-200b-3p expression in the basal TNBC compared to ER^+^/epithelial cancer cell lines, in addition we expand on this by showing miR-200b-5p is also equally reduced. In addition miR-200b-3p is unable to fully repress ZEB1 and ZEB2 suggesting that basal/TNBC have escaped the canonical ZEB/miR-200b feedback loop. Despite the lack of a large effect on ZEB1 and ZEB2, we demonstrate CDH1 repression and loss of invasive capacity in MDA-MB-231 –miR-200b cells. We suggest that this is through the fidelity in the targeting of miR-200b-3p and miR-200b-5p where both miRNAs target the RHOGDI signaling pathway. Non-canonical targeting of the RHO pathway by miR-200b-3p has also been recently demonstrated [14]. We expanded on these findings by demonstrating alterations in the RHOGDI signaling pathway correlate with a TNBC phenotype in clinical samples.

miR-200b-3p can directly target the 3ʹUTR of PRKCA and inhibition of metastasis of TNBC, supporting our findings [19]. Additionally, PRKCA can activate RHOGDI, which in turn activates RHOA, illustrating the importance of PRKCA and RHO signaling in the progression and metastasis of TNBC. Our data indicates that miR-200b-5p may also directly target PRKCA, while miR-200b-3p is predicted to target RHOA in addition to PRKCA. Figure 5B illustrates the predicted miR-200b-3p and miR-200b-5p targeting of the RHO signaling pathway at multiple points, with down regulated genes indicated in blue. The importance of these findings demonstrates novel mechanisms for the intervention of TNBC.

## MATERIALS AND METHODS

### Cells and reagents

MCF-7, ZR75, MDA-MB-157, MDA-MB-231, Hs578t, and BT-549 human breast cancer cell lines were acquired from American Type Culture Collection (Manassas, VA). Liquid nitrogen stocks were made upon receipt and maintained until the start of study. Morphology and doubling times were also recorded regularly to ensure maintenance of phenotype for all cell lines. Cells were used for no more than 6 months after being thawed. Cells were maintained as previously described [56]. Briefly cells were maintained at 37°, 5% CO2 in DMEM (Invitrogen, Carlsbad, CA, USA) supplemented with 10% fetal bovine serum (Hyclone, Salt Lake City, UT, USA) and 1% penicillin/streptomycin (Invitrogen).

### RNA extraction and quantitative

*Real Time RT-PCR* MDA-MB-231-vector and MDA-MB-miR-200b cells were harvested for total RNA extraction using Qiagen RNeasy RNA purification system or for microRNA miRNeasy purification system per manufacturer's protocol (Qiagen, Valencia, CA). Quantity and quality of the RNA and miRNA were determined by absorbance at 260 and 280 nm using the NanoDrop ND-1000. 2ug of total RNA was reverse-transcribed using the iScript kit (BioRad Laboratories, Hercules, CA) and qPCR was performed using SYBR-green (Bio-Rad Laboratories, Hercules, CA). β-Actin, CDH1, PRKCA, ZEB1, and ZEB2 amplified *n* > 3. miRNA was reverse–transcribed using the SABiosciences RT2 miRNA first strand kit (Qiagen, Valencia, CA) and qPCR was performed using SABiosciences SYBR green, miR-200b-3p, miR-200b-5p, and U6 primer purchased from Qiagen (Valencia, CA). Data was analyzed by comparing relative target gene expression to β-actin for mRNA and U6 for miRNA. Relative gene expression was analyzed using 2-ΔΔCt method [57].

### Transfection of cell lines

pri-miR-200b and empty vector pLemir plasmids were purchased from Open Biosystems (Lafayette, CO). MDA-MB-231 cell line was transfected through lenti-viral transfection as previously described [58] and retrovirus packing was performed following the manufacturer's instructions (Thermo ScientificBio, Pittsburgh PA). The following day cells were treated with 300 ng/ml puromycin. Cells were maintained in 10% DMEM and treated with 300 ng/ml puromycin every two days for 2 weeks. Colonies were pooled and verification of mature miR-200b-3p and later -5p overexpression was confirmed using qPCR for mature miR-200b-3p and -5p. Stable pools were maintained in 10% DMEM as described above.

### RNA-sequencing analysis

Read preparation, repeat masking, and read mapping were conducted as we have previously published [59]. In addition to our published methods, reads were mapped using custom perl scripts. A read is considered mapped to a gene if all but 2 (or fewer) bases of the read map to annotated exons of a gene (though the exon boundaries do not have to correspond to an identified isoform). After mapping the collapsed read set was expanded back to its original size both so that the disposition for every read could be accounted for and to facilitate the use of other tools expecting a standard SAM formatted dataset. Counts were determined from the expanded set of reads. Typically a read was mapped to one and only one annotated element in the human genome. While unlikely due to the strandedness of the reads, a read that was mapped to multiple genes in the genome incremented the count for each gene. Reads that map to multiple locations were marked as ambiguous and were not counted. Reads that were marked as repetitive (due to RepeatMasker) were used only if they could be mapped to a unique location in the genome.

Differential gene expression was determined using the edgeR software [60] [version 2.6.0] by supplying it the raw gene counts. Dispersion was estimated using both the estimatecommonDisp and estimateTagwiseDisp methods [61, 62]. A prior *n* value = 10 was used for running estimateTagwiseDisp. The exactTest method was run using default parameters allowing edgeR to decide which dispersions to use [63]. Pathway analysis was performed using GeneGo Metacore (Thomson Reuters). The Enrichment Analysis Workflow was performed using the gene list, fold-change and *p*-value scores generated by edgeR. A threshold *p*-value of < 0.05, and threshold fold-change < 0.5 was set when performing the analysis in GeneGo.

Data derived from RNA sequencing were analyzed for pathway analysis through the use of QIAGEN's Ingenuity® Pathway Analysis (IPA®, QIAGEN Redwood City, www.qiagen.com/ingenuity).

### Transwell migration assay

Migration assays were performed following the manufacturer's instructions (BD Biosciences, San Jose, CA) and as previously published [64]. Cells were seeded at a density of 2.5 × 10^4^ cells per well in serum free media. DMEM supplemented with 10% FBS (10%) was used as a chemo-attractant. DMEM without FBS (0%) served as a negative control. After 24 hours, migrated cells were fixed to the membranes and stained. Migrated cells visualized by microscopy, and data is represented as number of migrated cells per field of view ± SEM for triplicate experiments.

### Breast cancer data sources

Breast cancer gene expression deep sequencing was viewed through the University California Santa Cruz (UCSC) Cancer Genomics Browser and compiled by The Cancer Genome Atlas (TCGA) research network [46–49]. The TCGA dataset used was the breast invasive carcinoma and it was analyzed for gene expression aligned through the Illumina HiSeq system total tumor samples were *n* = 1032.

### Statistical analysis

Statistical Analysis was performed using Graph Pad Prism 5. Student's *t*-test was used to determine *p* values and statistically significant values had a *p* values of < 0.05.

## SUPPLEMENTARY FIGURE AND TABLE


