# Supplementary material for: Dual regulation by microRNA-200b-3p and microRNA-200b-5p in the inhibition of epithelial-to-mesenchymal transition in triple-negative breast cancer
Source: Oncotarget. 2015 Mar 21;6(18):16638–52. doi: 10.18632/oncotarget.3184 (PMC4599295; doi:10.18632/oncotarget.3184)
Supplement: Supplementary file 1 [file oncotarget-06-16638-s001.pdf]

SUPPLEMENTARY FIGURE AND TABLE

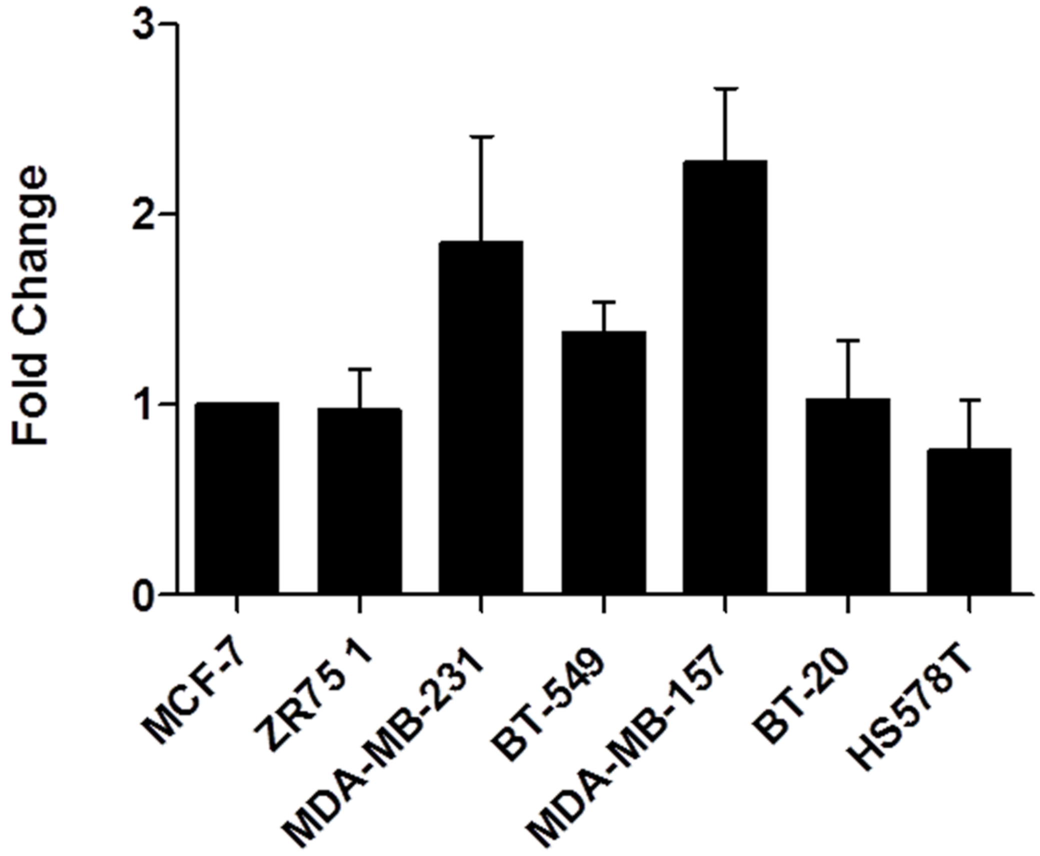

Supplemental Figure 1: PRKCA Expression Across ER+ and Triple Negative Breast Cancer Cell Lines. A cohort of ER+ (ZR75, MCF-7) and ER- (MDA-MB-157, MDA-MB-231, BT-549, Hs578t) breast cancer cell lines were tested by qPCR to determine basal PRKCA expression. B-actin was used for internal normalization and compared to the MCF-7 breast cancer cell line (designated as 1).

Supplemental Table 1: miR-200b expression in MDA-MB-231 cell line induces gene expression changes in genes associated with EMT

Gene Name	LogFc	*p*-value	FDR
CDH1	2.03	8.12E-10	7.88E-08
CDH2	4.73	7.88E-42	1.44E-38
COL1A2	–2.44	3.08E-32	2.61E-29
EGFR	0.39	0.000129	0.003723
ERBB3	1.44	2.31E-11	2.83E-09
F11R	0.95	5.17E-14	9.25E-12
FN1	–0.51	1.19E-06	6.15E-05
IGFBP4	–0.70	2.83E-07	1.69E-05
ITGB1	–0.64	1.48E-11	1.87E-09
KRT19	0.57	7.05E-05	0.00223
KRT7	–0.75	1.32E-07	8.53E-06
MITF	0.70	0.001347	0.026478
MST1R	0.77	1.95E-08	1.44E-06
OCLN	1.92	1.62E-13	2.68E-11
PDGFRB	–2.13	6.46E-08	4.38E-06
SERPINE1	1.12	1.25E-15	2.69E-13
TMEM132A	0.54	0.002551	0.043995
TSPAN13	2.19	4E-06	0.000182
WNT5B	0.51	0.000411	0.009961
VIM	–0.16	0.17978	0.873841
ZEB1	–0.97	6.87E-12	8.87E-10
ZEB2	–0.72	0.001772	0.032987
